# Optical gradient and translucency behavior of three multilayer monolithic zirconia systems: a spectrophotometric study

**DOI:** 10.1038/s41598-026-50564-5

**Published:** 2026-04-27

**Authors:** Özlem Özişçi

**Affiliations:** Department of Prosthodontics, Faculty of Dentistry, Süleyman Demirel University, Isparta, Turkey

**Keywords:** CAD-CAM restorations, Monolithic ceramics, Shade matching, Spectrophotometry, Health care, Materials science, Medical research, Optics and photonics

## Abstract

This in vitro study evaluated the effects of multilayer zirconia material, anatomical region, substrate shade, and laboratory background conditions on the optical behavior of monolithic zirconia crowns. Sixty crowns fabricated from three multilayer zirconia systems were analyzed at cervical, middle, and incisal regions using standardized white, black, and neutral backgrounds. Color coordinates (L*, a*, b*), color difference (ΔE₀₀), translucency parameter (TP₀₀), and contrast ratio (CR) were measured using a dental spectrophotometer. Material type and anatomical region significantly influenced all optical parameters. Perfit STML demonstrated higher translucency (TP₀₀ ≈ 1.50) and color difference (ΔE₀₀ ≈ 3.71), whereas Dental Direkt exhibited lower translucency (TP₀₀ ≈ 0.99) and higher opacity (CR ≈ 0.98). The incisal region showed the highest translucency (TP₀₀ ≈ 2.33) and lowest contrast ratio, while the cervical region exhibited higher lightness and chromaticity. Laboratory background conditions affected color coordinates and ΔE₀₀ values, whereas intrinsic optical properties such as TP₀₀ and CR remained relatively stable across backgrounds. However, interaction analysis revealed that the influence of background conditions varied depending on the anatomical region. These findings indicate that multilayer zirconia materials are not optically interchangeable and that their optical performance is strongly influenced by both material composition and anatomical location. Clinically, highly translucent materials may be preferred for achieving a natural incisal appearance under favorable substrate conditions, whereas more opaque materials or masking strategies may be required when the underlying substrate is discolored.

## Introduction

The growing demand for esthetic dental restorations has driven significant advances in all ceramic materials, which offer improved biocompatibility and superior mimicry of natural teeth compared with conventional metal-ceramic restorations^1,2^. Among these all ceramic materials, zirconia has become exceptionally popular due to its excellent mechanical properties, including high flexural strength and fracture toughness, making it suitable for a wide range of clinical applications from single crowns to multi-unit fixed dental prostheses^3^.

The evolution of zirconia ceramics has seen a shift from the early, highly opaque frameworks used in bilayered restorations to more translucent, monolithic materials^2,4^. This progression was largely in response to the clinical complication of veneer chipping associated with porcelain fused to zirconia restorations^4,5^.

The latest generation of materials includes highly translucent and ultra-translucent multilayered zirconia blocks, designed with a gradient in shade and translucency from the cervical to the incisal region to better replicate the natural polychromaticity of a tooth^6,7^. These materials aim to provide durable, single structure restorations with lifelike esthetics, making them particularly promising for anterior teeth^8^. These multilayer systems are engineered through controlled gradients in yttria content, pigment distribution, and microstructure. Typically, higher yttria concentrations in the incisal region increase the cubic phase fraction and enhance translucency, whereas lower yttria levels in the cervical region preserve a predominantly tetragonal structure and contribute to higher opacity. Variations in pigment distribution and grain size across layers may further influence light scattering and color transitions within the restoration.

Despite these improvements, the increased translucency of modern zirconia presents a clinical challenge: the final perceived color of the restoration is significantly influenced by the underlying structures, including the color of the abutment tooth and the luting agent^1,2^. Discolored abutments or improperly selected cement shades can alter the final optical outcome, compromising the esthetic success of an otherwise well fabricated crown^9^. Several factors, including substrate shade, luting cement characteristics, and ceramic thickness, have been shown to influence the final color of zirconia restorations^1,10–16^.

To quantify these optical effects, dental research widely employs the Commission Internationale de l’Eclairage (CIE) L*a*b* color space, a three dimensional model where L* denotes lightness, a* represents the red-green axis, and b* represents the yellow-blue axis. Color differences between specimens are expressed using formulas such as CIELAB (ΔEab) or the more perceptually uniform CIEDE2000 (ΔE_00_), which can be compared against established perceptibility and acceptability thresholds to determine clinical relevance^2,4^. Furthermore, specific optical properties, including the translucency parameter, which measures the color difference of a material over white and black backgrounds, and opacity, often expressed as the contrast ratio, are used to characterize how materials transmit and scatter light^5,12,17–21^.

Although previous studies have evaluated the optical behavior of zirconia materials, most have focused on single layer systems or have examined optical and mechanical properties separately, while comprehensive analyses of multilayer zirconia systems under varying substrate and background conditions remain limited^4,5^.

Therefore, this in vitro study aimed to evaluate and compare the final color, translucency, and opacity of three different multilayer monolithic zirconia systems. The materials were selected using a purposeful sampling strategy to represent distinct multilayer zirconia design approaches currently available in clinical practice. Specifically, the selected systems differ in terms of yttria content distribution (3Y-5Y gradient), layer configuration, and translucency profiles. These systems were selected because they are widely used in clinical practice and represent different industrial formulations and yttria distributions within the current generation of high translucency zirconias. cubeX^2^ ML is designed to provide relatively higher opacity compared to ultra translucent systems, Katana YML incorporates a well documented multilayer yttria gradient (5Y-4Y-3Y) enabling a balance between translucency and strength, while Perfit STML represents a highly translucent multilayer zirconia with increased cubic phase content. This selection was intended to represent a clinically relevant range of translucency and microstructural variations influencing optical behavior.

The study assessed these optical properties when the crowns were placed against standardized white, black, and gray backgrounds, as well as substrates simulating A1, A2, and A3 tooth shades. The null hypothesis was that material type, anatomical region, substrate shade, and laboratory background conditions would not significantly influence the optical properties of the tested multilayer zirconia systems, and that no interaction effects would be observed among these variables.

## Materıal and methods

### Study design

**Fig. 1:**
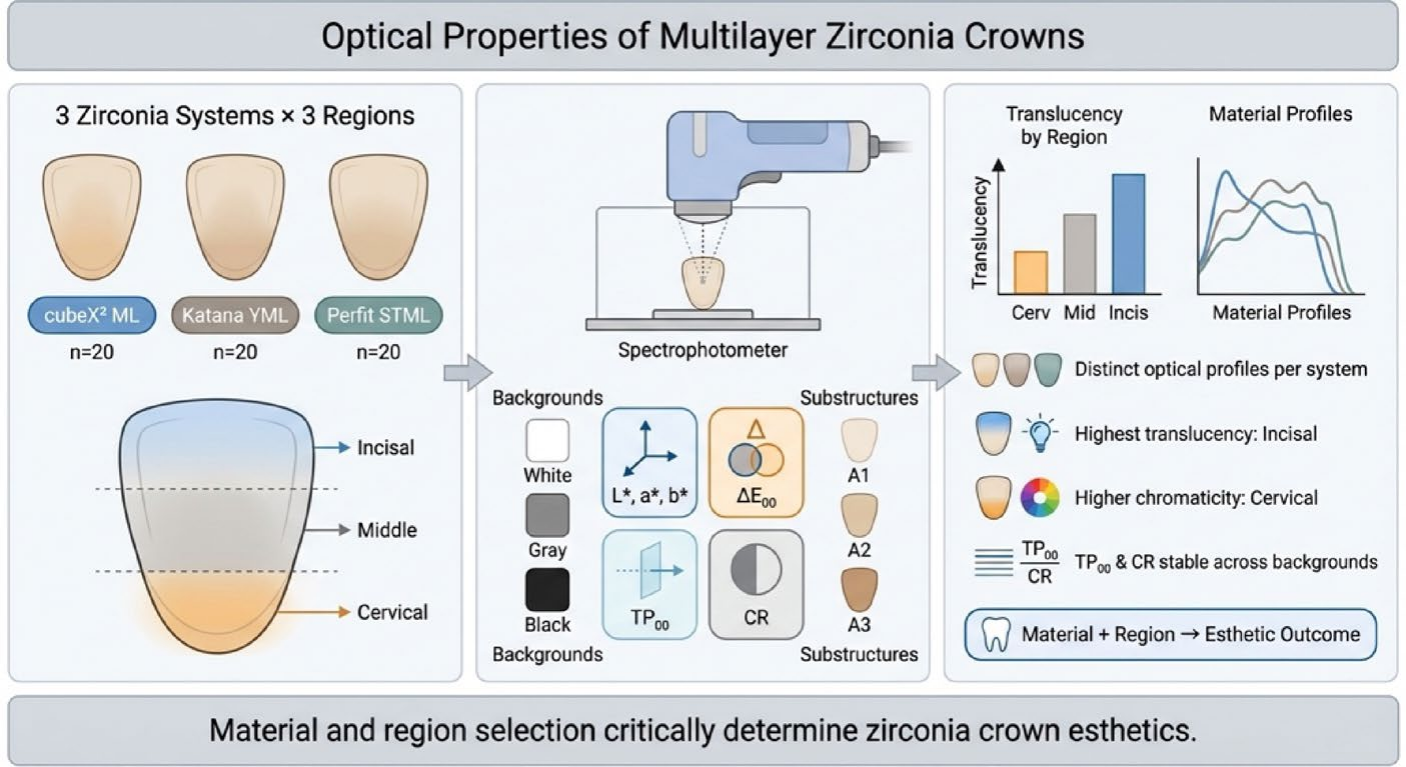
Overview of the study workflow and main optical analyses.

This in-vitro experimental study was conducted to evaluate the optical properties of three commercially available multilayer high translucency zirconia systems. The independent variables included zirconia material (three brands), anatomical measurement region (cervical, middle, and incisal), and substrate shade (A1, A2, and A3). The dependent variables were the CIELAB color coordinates (L*, a*, b*) and derived optical parameters: color difference (ΔE₀₀), translucency parameter (TP₀₀), and contrast ratio (CR), which were determined using spectrophotometry (Fig. 1). The main optical trends identified in the study were additionally presented in a combined graphical format (Fig. 3) to facilitate visual interpretation of the findings.

### Preparation of master die and substrate dies

An artificial maxillary left central incisor model (Frasaco GmbH, Tettnang, Germany) was prepared by a single operator to receive an all-ceramic crown. The preparation featured a 1.0-mm deep chamfer margin, an axial taper of approximately 6°, and an incisal reduction of 2.0 mm. An optical impression of the prepared abutment was captured using an intraoral scanner (TRIOS 5; 3Shape A/S, Copenhagen, Denmark) to generate the necessary standard tessellation language data. This digital scan served as the virtual master model for subsequent computer-aided design (CAD) and manufacturing of the crown, replacing the conventional polyvinyl siloxane impression and poured stone die method.

To simulate clinically relevant abutment tooth colors, the master die was duplicated using a nanohybrid universal composite resin (Filtek Z250; 3 M ESPE, St. Paul, MN, USA) in three distinct VITA Classical shades: A1, A2, and A3. Each composite duplicate was subsequently embedded in a transparent acrylic resin block to create stable and standardized substrate dies for the measurement protocol.

### Fabrication of zirconia crowns

The master die was digitized using a laboratory scanner (Dental Wings 7series; Straumann Group, Basel, Switzerland). Based on this scan data, a standardized full contour maxillary central incisor crown was then designed in CAD software (exocad DentalDB 3.1; exocad GmbH, Darmstadt, Germany). The design parameters included a uniform cement space of 50 µm, beginning 1.0 mm coronal to the finish line.

**Fig. 2:**
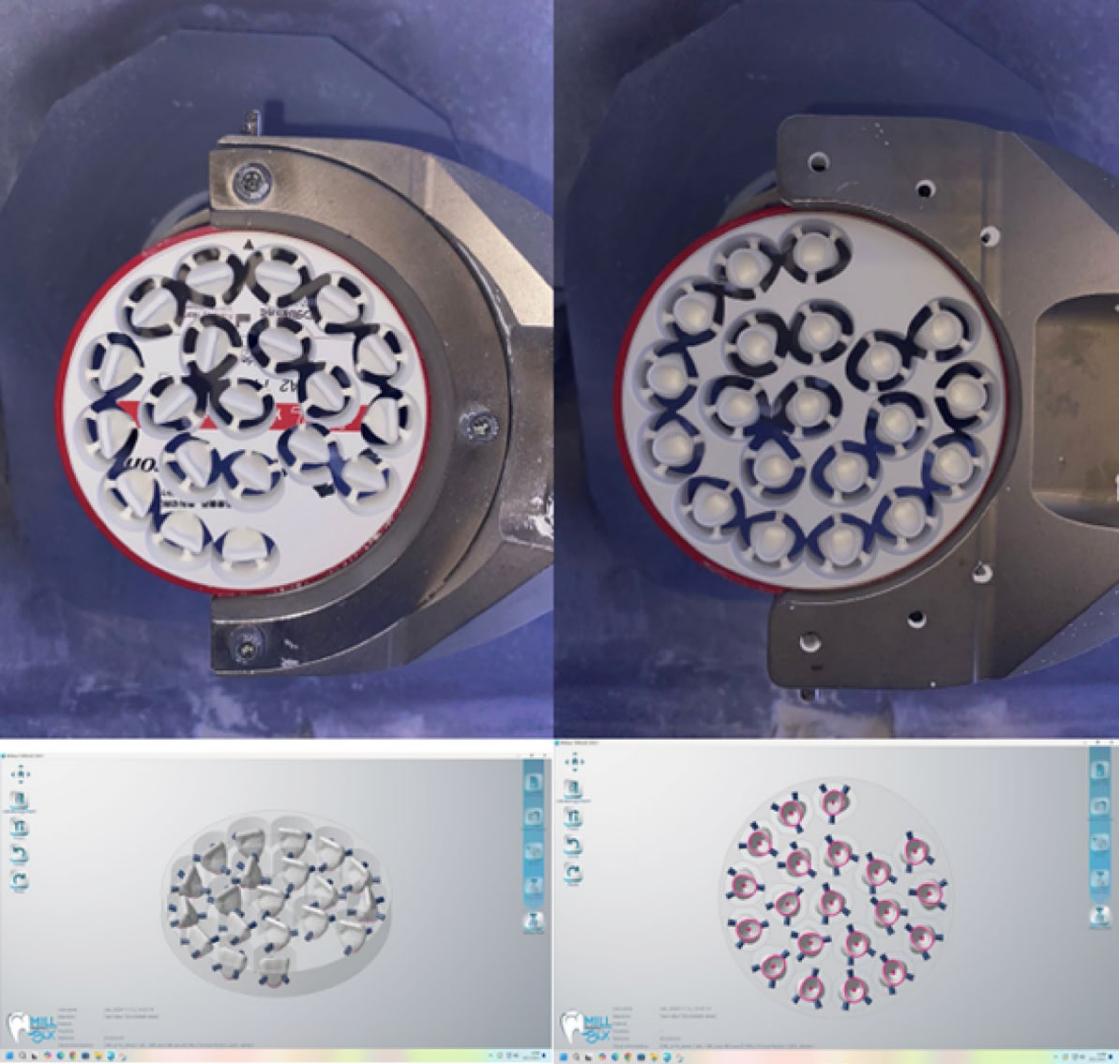
Digital design and milling workflow for multilayer zirconia crowns.

A total of 60 crowns (n = 20 per material group) were milled from multilayer high translucency zirconia blocks in the manufacturer designated A2 shade (Fig. 2).

Three multilayer zirconia systems were included to represent different categories of currently available high translucency zirconia materials with varying yttria content distributions and optical characteristics.

The three zirconia systems evaluated were:

- Dental Direkt cubeX^2^ ML (Dental Direkt GmbH, Spenge, Germany)
- Katana YML (Kuraray Noritake Dental Inc., Tokyo, Japan)
- Perfit STML (Perfit Dental, Nanjing, China)

Material characteristics, including yttria content gradients, phase composition, and layer structure, were obtained from manufacturer provided technical data and are summarized in Table 1. Following milling, all crowns were sintered in a furnace according to each manufacturer’s specific recommendations for time and temperature. To ensure procedural consistency, an experienced dental technician then applied glaze and performed the final polishing on all 60 specimens. Final thicknesses of each crown at the cervical, middle, and incisal regions were measured using a digital caliper with an accuracy of ± 0.01 mm.

**Table 1:** Characteristics of the zirconia materials evaluated in the study.

Material (manufacturer)	Layer structure	Yttria content (mol% Y₂O₃)	Phase composition	Layer thickness distribution	Grain size / microstructure	Additional notes
Dental Direkt cubeX^2^ ML (Dental Direkt GmbH, Germany)	Multilayer	≈ 4–5 (limited manufacturer data)	Mixed tetragonal + cubic	Multilayer gradient	Not specified by manufacturer	High translucency,multilayer gradient
Katana YML (Kuraray Noritake, Japan)	4-layer system (Enamel/ Transition/ Dentin/ Cervical)	Enamel: ≈5Y Dentin: ≈4Y Cervical: ≈ 3Y	Incisal: cubic-rich, Cervical: tetragonal-dominant	Progressive gradient from incisal to cervical (incisal more translucent)	Fine-grained structure (manufacturer data)	Designed for natural optical gradient
Perfit STML (Perfit Dental, China)	Multilayer	≈ 4–5 (gradient not fully disclosed by manufacturer)	Predominantly tetragonal with partial cubic phase	Multilayer color gradient	Not specified	Super translucent multilayer zirconia

*Where detailed layer-specific data were not disclosed by manufacturers, this limitation has been explicitly stated to avoid overinterpretation of compositional differences.*

### Spectrophotometric analysis and measurement protocol

For clarity, two different optical conditions were defined in this study. The term “substrate shade” refers to the color of the composite-acrylic dies (A1, A2, and A3), which simulate the underlying abutment tooth. The term “background” refers to the standardized laboratory background plates (white, black, and gray) used for translucency and contrast measurements. These terms are used consistently throughout the manuscript to avoid ambiguity. All colorimetric measurements were performed by a single calibrated operator inside a custom-made light box equipped with standardized D65 daylight illumination to ensure consistent viewing conditions. A calibrated dental spectrophotometer (VITA Easyshade V; VITA Zahnfabrik, Bad Säckingen, Germany) was used to record the CIE L*a*b* color coordinates. A custom fabricated jig was employed to ensure precise and repeatable positioning of the spectrophotometer’s probe on the flattest area of the labial surface for each measurement. Each of the 60 zirconia crowns was evaluated under multiple substrate shade and laboratory background conditions. To assess the influence of the underlying shade, each crown was seated on composite acrylic substrate dies in shades A1, A2, and A3.

A thin layer of glycerin was applied as a coupling medium between the crown and each substrate to minimize interfacial light scattering without introducing the additional color variability associated with resin cements. Resin cements exhibit a wide range of shades, opacities, and polymerization related optical changes that can substantially influence the final color of thin ceramic restorations. Because the primary aim of this study was to isolate the contributions of zirconia material, anatomical region, and background shade to ΔE₀₀, TP₀₀, and CR, cement effects were intentionally excluded. This approach improves internal validity for the studied factors, while acknowledging that cement shade remains critical in clinical shade matching. However, this does not fully replicate clinical conditions where resin-based luting agents are used.

For translucency and opacity calculations, measurements were also taken against three standardized backgrounds: neutral gray (L* ≈ 50), white (L* ≈ 98.1), and black (L* ≈ 4.7).

Color coordinates were recorded from three distinct anatomical regions on the labial surface of each crown:

- Cervical: The collar region, located 1.0 mm coronal to the margin.
- Middle: The central third of the labial surface.
- Incisal: The incisal third of the labial surface.

For every condition (e.g., a specific crown on an A2 substrate measured at the middle region), three consecutive readings were obtained. The crown was removed and repositioned in the jig between readings to enhance measurement reliability. The three repeated measurements obtained under each condition were averaged to produce a single representative value per specimen. Therefore, the statistical analysis was performed using these mean values, and repeate measures analysis was not required.

### Calculation of optical properties

From the spectrophotometric CIELAB data, ΔE₀₀, TP₀₀, and CR were calculated. Color difference was determined using the CIEDE2000 formula.

$$\Delta E_{00} = \sqrt {\left( {\frac{{\Delta L^{\prime } }}{{K_{L} S_{L} }}} \right)^{2} + \left( {\frac{{\Delta C^{\prime } }}{{K_{C} S_{C} }}} \right)^{2} + \left( {\frac{{\Delta H^{\prime } }}{{K_{H} S_{H} }}} \right)^{2} + R_{T} \left( {\frac{{\Delta C^{\prime } }}{{K_{C} S_{C} }}} \right)\left( {\frac{{\Delta H^{\prime } }}{{K_{H} S_{H} }}} \right)}$$

The translucency parameter was calculated from the measurements obtained against the standard white and black backgrounds.

$$TP_{00} = \sqrt {\left( {\frac{{L_{B}^{\prime } - L_{W}^{\prime } }}{{K_{L} S_{L} }}} \right)^{2} + \left( {\frac{{C_{B}^{\prime } - C_{W}^{\prime } }}{{K_{C} S_{C} }}} \right)^{2} + \left( {\frac{{H_{B}^{\prime } - H_{W}^{\prime } }}{{K_{H} S_{H} }}} \right)^{2} + R_{T} \left( {\frac{{C_{B}^{\prime } - C_{W}^{\prime } }}{{K_{C} S_{C} }}} \right)\left( {\frac{{H_{B}^{\prime } - H_{W}^{\prime } }}{{K_{H} S_{H} }}} \right)}$$

The contrast ratio was derived from the tristimulus Y values (luminance), which were calculated from the L* values measured against the black (Yb) and white (Yw) backgrounds.

### Sample size calculation

An a priori sample size estimation was performed using G*Power software (Heinrich Heine University Düsseldorf, Düsseldorf, Germany) to support the experimental design. The calculation was based on an F-test model for fixed effects and interactions, considering the factorial structure of the study. An alpha error probability of 0.05 and a statistical power of 0.80 were assumed, with a medium effect size (f = 0.25). The assumed medium effect size (f = 0.25) was selected based on previously reported differences in optical parameters of multilayer zirconia systems in the literature. Prior studies evaluating translucency (TP₀₀) and color difference (ΔE₀₀) have reported measurable between material differences corresponding to at least moderate effect magnitudes in factorial designs^22,23^. In these studies, the magnitude of between material differences in optical parameters has been reported to correspond to moderate to large effect sizes, with partial eta squared (η^2^) values typically ranging between approximately 0.10 and 0.25. Therefore, the assumption of a medium effect size (f = 0.25) is methodologically justified and consistent with previously reported clinically meaningful differences in zirconia translucency.

Based on these parameters, a sample size of 20 crowns per material group was considered sufficient to detect both main and interaction effects among material type, anatomical region, and laboratory background conditions. However, this estimation is primarily valid for medium to large effect sizes, and smaller interaction effects may require larger sample sizes for more precise detection.

### Statistical analysis

All statistical analyses were performed using SPSS software (version 26.0; IBM Corp., Armonk, NY, USA), with the significance level set at α = 0.05. Descriptive statistics (mean and standard deviation) were calculated for all optical parameters, and normality of data distribution was assessed using the Shapiro–Wilk test.

The primary inferential analysis was conducted using a full-factorial general linear model (GLM) to evaluate the main effects of material type, anatomical region, substrate shade, and laboratory background conditions, as well as their interaction effects. Effect sizes were calculated and reported as partial eta squared (partial η^2^) to assess the magnitude of the observed effects. This approach was selected because it allows simultaneous evaluation of multiple independent variables and their interaction effects within a single statistical model.

One-way ANOVA was performed only for descriptive, factor specific comparisons to facilitate interpretation of group level differences. However, these analyses were not used for inferential conclusions. All primary statistical inferences were based on the full factorial GLM, which accounts for the multifactorial structure and interaction effects of the study design.

Post hoc multiple comparisons were performed using Bonferroni adjustment to control for Type I error. Duncan’s multiple range test, used in earlier stages of the analysis, was not included in the final statistical evaluation due to its relatively liberal nature and potential to increase Type I error rates.

Pearson correlation analysis was performed to assess relationships among the evaluated optical parameters.

Because repeated measurements were averaged prior to analysis, each specimen contributed a single representative value per condition. Therefore, the statistical analyses were conducted using independent observations.

## Results

All optical parameters exhibited normal distribution, supporting the use of parametric statistical analyses. Effect size analysis further indicated that based on partial eta squared (η^2^) indicated that material type and anatomical region exerted moderate to large effects on the evaluated optical parameters, whereas interaction effects were generally smaller in magnitude.

Material type significantly influenced color coordinates and derived optical parameters. Dental Direkt demonstrated the highest lightness values and slightly more positive a* values, indicating a relatively brighter and more reddish appearance. Katana YML exhibited the highest b* values, reflecting increased yellow chromaticity. In contrast, Perfit STML showed more negative a* values and higher color difference values compared with the other materials. Considering the perceptibility threshold (ΔE₀₀≈1.18), Perfit STML exhibited a higher proportion of values exceeding this limit, particularly in the incisal region. Clinically, this suggests that material selection based solely on shade designation may be insufficient, and intrinsic optical behavior must be taken into account.

Perfit STML demonstrated higher translucency and lower contrast ratio values, whereas Dental Direkt exhibited lower translucency and relatively higher opacity (Table 2). The difference in translucency between Perfit STML (TP₀₀≈1.50) and Dental Direkt (TP₀₀≈0.99) corresponds to an approximate 50% increase in light transmission, representing a clinically meaningful difference. Increased translucency may enhance restoration vitality but can reduce masking ability. Therefore, material selection should be carefully considered, particularly in cases involving discolored substrates.

**Table 2:** Comparison of mean (± SD) optical properties among zirconia materials.

Optical Parameter	Dental Direkt	Perfit STML	Katana YML	*p*-value
L* (Lightness)	86.96 (± 1.09) ᵃ	83.03 (± 2.71) ᵇ	81.74 (± 1.38) ᶜ	*p* < 0.001
a* (Red-Green)	0.17 (± 0.28) ᵃ	− 1.62 (± 0.85) ᶜ	− 0.38 (± 1.02) ᵇ	*p* < 0.001
b* (Yellow-Blue)	25.19 (± 3.39) ᵇ	24.92 (± 5.66) ᵇ	25.92 (± 6.61) ᵃ	*p* < 0.001
**ΔE₀₀ (Color Difference)**	1.06 (± 0.61) ᵇ	3.71 (± 4.13) ᵃ	1.10 (± 0.83) ᵇ	*p* < 0.001
**TP₀₀ (Translucency)**	0.99 (± 0.78) ᶜ	1.50 (± 1.25) ᵃ	1.33 (± 1.01) ᵇ	*p* < 0.001
**CR (Contrast Ratio)**	0.98 (± 0.04) ᵃ	0.97 (± 0.05) ᵇ	0.98 (± 0.05) ᵃ	*p* < 0.001

Within each row, different superscript letters (a, b, c) denote statistically significant differences between groups (p < 0.05)

**Fig. 3:**
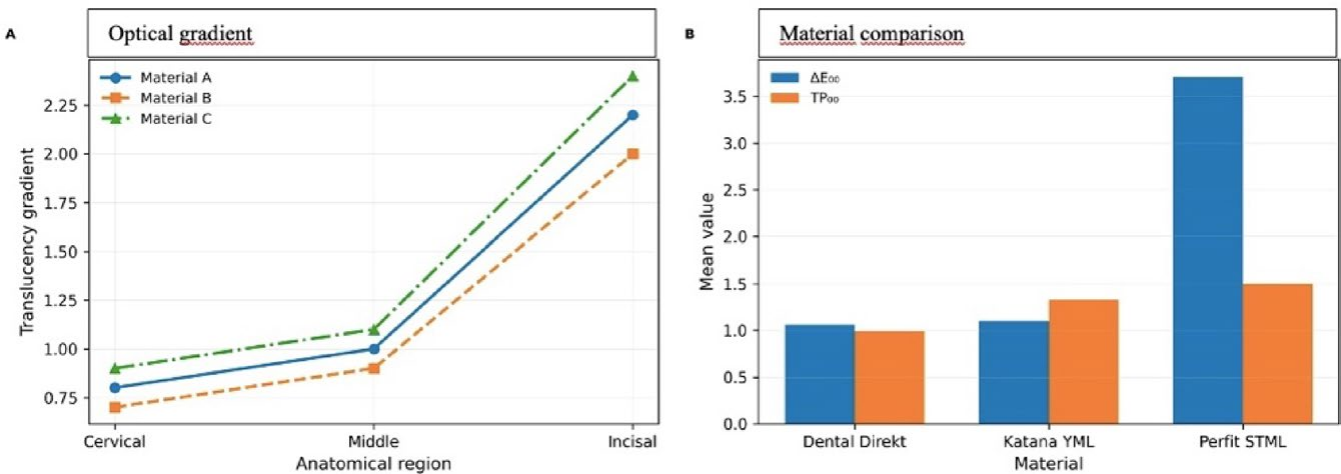
(**A**) Schematic illustration of the optical gradient across cervical, middle, and incisal regions in multilayer zirconia. (**B**) Graphical comparison of mean optical parameters among the evaluated materials.

The anatomical measurement region influenced all optical parameters (Table 3). The cervical region showed higher lightness and stronger yellow–red chromaticity compared with the middle and incisal regions. In contrast, the incisal region exhibited lower lightness and chromatic values but higher translucency values and lower contrast ratios, indicating greater optical transparency. These findings are further illustrated in Fig. 3A, which schematically demonstrates the gradual optical transition from the cervical region to the incisal region. These findings confirm the presence of a gradient optical behavior within multilayer zirconia systems. Clinically, this suggests that the incisal region represents the most esthetically sensitive area, where minor variations in material properties or substrate conditions may significantly affect the final appearance.

**Table 3:** Comparison of mean (± SD) optical properties among anatomical regions.

Optical parameter	Cervical region	Middle region	Incisal region	*p*-value
L* (Lightness)	84.63 (± 2.64) ᵃ	83.09 (± 2.72) ᶜ	84.01 (± 3.11) ᵇ	*p* < 0.001
a* (Red-Green)	− 0.03 (± 0.71) ᵃ	− 0.32 (± 0.65) ᵇ	− 1.47 (± 1.19) ᶜ	*p* < 0.001
b* (Yellow-blue)	31.12 (± 2.32) ᵃ	26.04 (± 1.40) ᵇ	18.86 (± 2.08) ᶜ	*p* < 0.001
ΔE₀₀ (Color difference)	1.79 (± 2.61) ᵇ	1.72 (± 2.74) ᵇ	2.35 (± 2.86) ᵃ	*p* < 0.001
TP₀₀ (Translucency)	0.74 (± 0.53) ᵇ	0.76 (± 0.40) ᵇ	2.33 (± 1.10) ᵃ	*p* < 0.001
CR (Contrast ratio)	1.00 (± 0.03) ᵃ	0.99 (± 0.02) ᵇ	0.93 (± 0.05) ᶜ	*p* < 0.001

*Within each row, different superscript letters (a, b, c) denote statistically significant differences between groups (p* < *0.05).*

Laboratory background conditions affected the measured color coordinates and ΔE₀₀ values. Measurements obtained against white backgrounds showed higher L*, a*, and b* values compared with darker backgrounds. In contrast, TP₀₀ and CR values remained relatively stable across different background conditions. This indicates that intrinsic optical parameters such as translucency and opacity are less influenced by external measurement conditions. Clinically, however, background related effects remain relevant for color perception, particularly in highly translucent regions.

Interaction analysis revealed that the influence of laboratory background conditions on color difference varied depending on the anatomical region. In addition, the relationship between anatomical region and optical behavior differed among zirconia materials, indicating material-dependent optical gradients. These findings demonstrate that optical performance is governed by complex interactions rather than by single variables alone. Clinically, this highlights the importance of considering both material properties and anatomical factors simultaneously when planning esthetic restorations.

Analysis of perceptibility thresholds showed that Perfit STML had a lower proportion of samples below the perceptibility limit compared with Dental Direkt and Katana YML. The incisal region exhibited the lowest proportion of imperceptible color differences, whereas measurements obtained against white backgrounds resulted in fewer imperceptible matches. This suggests that highly translucent materials and incisal regions are more prone to clinically perceptible color discrepancies, increasing the risk of esthetic mismatch under unfavorable substrate conditions.

Correlation analysis revealed a strong inverse relationship between TP₀₀ and CR. In addition, ΔE₀₀ showed a weak positive correlation with TP₀₀ and a weak negative correlation with CR (Table 4). These relationships confirm that increasing translucency is inherently associated with reduced opacity and increased sensitivity to background influence. Clinically, this trade-off must be carefully managed to achieve an optimal balance between esthetic integration and masking ability.

**Table 4:** Pearson correlation matrix for optical properties.

	**ΔE₀₀**	**TP₀₀**	**CR**
ΔE₀₀	1		
TP₀₀	0.265*	1	
CR	− 0.218*	− 0.791*	1

*Correlation is significant at the 0.05 level.

Overall, the results demonstrate that the optical behavior of multilayer zirconia systems is governed by multifactorial interactions involving material composition, anatomical region, and environmental conditions. These findings clearly reject the null hypothesis and emphasize that optical performance cannot be predicted based on a single parameter alone.

## Dıscussıon

The increasing demand for highly esthetic monolithic restorations has stimulated the development of multilayer zirconia materials designed to reproduce the natural chromatic and translucency gradients of teeth^13,14^. The present in vitro study therefore aimed to evaluate the optical behavior of three multilayer zirconia systems under different anatomical, substrate, and laboratory background conditions. The findings demonstrated that these systems exhibit distinct and material dependent optical profiles, confirming that multilayer zirconia materials with the same nominal shade designation cannot be assumed to behave identically.

A primary finding of this study was that the three evaluated zirconia materials (Dental Direkt, Katana YML, and Perfit STML) exhibited distinct and statistically different optical profiles. Dental Direkt demonstrated higher lightness values and a slightly more reddish tendency, whereas Katana YML showed greater yellow chromaticity. In contrast, Perfit STML exhibited a more greenish tendency together with higher translucency. Rather than representing only numerical differences, these material-dependent optical characteristics suggest that multilayer zirconia systems should be selected according to the specific esthetic requirements of the restoration. However, because detailed manufacturer-specific data on pigment concentration, grain size, and exact phase distribution were not fully available, these explanations should be interpreted as plausible rather than definitive^15,16^.

The observed differences in ΔE₀₀ and TP₀₀ values indicate a more optically active structure in the more translucent material. This may enhance restoration vitality. However, it may also complicate color matching when the underlying substrate shade is suboptimal. Figure 3B further illustrates the material dependent optical differences. These findings are consistent with recent investigations evaluating multilayer zirconia systems with different yttria compositions and thicknesses. Kang et al. reported that multilayer zirconia systems combining 4Y and 5Y yttria levels exhibit higher translucency (TP₀₀) values compared to conventional 3Y zirconia, while demonstrating reduced fracture loads, indicating a trade-off between optical performance and mechanical strength^22^. In the present study, a similar trend was observed, where Perfit STML, characterized by higher translucency, showed increased TP₀₀ and ΔE₀₀ values which is consistent with the optical trends reported for zirconia systems with higher yttria content.

Beyond compositional differences, structural factors such as restoration thickness also influence optical behavior. Studies evaluating the effect of restoration thickness have demonstrated a strong inverse relationship between thickness and translucency, alongside a positive correlation with fracture resistance. Kang et al.^23^ reported that decreasing crown thickness significantly increases translucency while reducing fracture strength, with incisal regions exhibiting the highest translucency values due to reduced material thickness and structural differences. These findings are in agreement with the present study, where the incisal region demonstrated higher TP₀₀ values and greater sensitivity to laboratory background conditions, confirming its optical and clinical significance.

The study also confirmed that modern MLZ blocks successfully replicate the optical gradient of natural teeth^9,17^. The incisal region exhibited higher translucency (higher TP₀₀, lower CR) than the middle and cervical regions, consistent with the natural tooth structure, where the enamel is more translucent at the incisal edge. Conversely, the cervical region was the most opaque and displayed the highest yellow–red chromaticity, reflecting the characteristic of cervical dentin^1^. This structural gradient underscores the potential of MLZ for anterior restorations, aligning with existing literature that links the degree of opacity to the tooth’s underlying dentin characteristics^1,15^. This gradient pattern is also visually supported by the schematic illustration shown in Fig. 3A. Although the present study focused on optical behavior, the observed increase in translucency toward the incisal region may also have biomechanical implications, as thinner and more translucent regions may exhibit reduced fracture resistance, as previously reported.

The most important findings relate to the interaction effects identified by the GLM analysis. The significant interaction between material and anatomical region indicates that the optical gradient is material dependent. Therefore, material selection should be tailored to the esthetic demands of each case. Some systems provide a subtle transition, whereas others exhibit a more pronounced shift from cervical to incisal regions.

From a practical standpoint, highly translucent multilayer zirconia systems may be more suitable in cases with favorable substrate shades and high esthetic demands, particularly when a natural incisal effect is desired. In contrast, materials with lower translucency and greater masking ability may be preferable in cases involving discolored substrates. In such cases, clinicians should consider modifying restoration thickness and contour. In particular, increasing incisal thickness may help reduce excessive background influence while maintaining an acceptable esthetic gradient.

Furthermore, the significant interaction between Region and laboratory background conditions for ΔE₀₀ underscores a critical clinical challenge. The influence of the underlying substrate (abutment tooth and cement) varies across the crown, being more pronounced in highly translucent areas, such as the incisal region, and less so in opaque areas like the cervical region. This finding corroborates previous research emphasizing the effect of substrate color on the esthetic outcome of translucent restorations^1,11,18^. Accordingly, when restoring a discolored tooth, the masking capacity of the cervical portion of a multilayer crown may be sufficient, but the translucent incisal third may fail to adequately block the dark underlying shade, potentially leading to an esthetic failure^19^. In these situations, clinicians may need to combine careful material selection with restorative design modifications, such as increasing incisal thickness where possible, selecting a less translucent zirconia system, or using additional masking strategies at the substrate or cement level. Such decisions are particularly relevant in anterior restorations, where the esthetic demands are high and the incisal region is the most optically sensitive.

The analysis of laboratory background conditions on optical measurements also yielded important methodological insight. Although TP₀₀ and CR values were not directly influenced by background conditions, interaction analysis revealed that the effect of background on optical behavior varied depending on the anatomical region^20,21^. This indicates that the translucency parameter and contrast ratio are robust, background-independent parameters suitable for characterizing the inherent optical properties of a material under controlled laboratory settings. In contrast, the context dependency of L*a*b* values reinforces the need to consider the complete restorative complex for reliable clinical shade matching.

From a clinical perspective, perceptibility thresholds analysis provides practical guidance. The incisal region exhibited the highest proportion of perceptible color differences (i.e., the lowest percentage below threshold), confirming it as the most esthetically sensitive zone. Similarly, the more translucent material showed a higher proportion of perceptible color differences, consistent with increased light transmission and greater susceptibility to background influence.

This study has several limitations. First, this was an in‑vitro study conducted under controlled laboratory conditions without simulation of aging or intraoral challenges. No thermocycling, mechanical loading, or exposure to saliva, pigments or pH fluctuations was applied; these factors may alter the optical properties of zirconia and its interfaces over time and could lead to different ΔE₀₀ and TP₀₀ values in vivo. Second, all spectrophotometric measurements were performed by a single, calibrated operator using a standardized jig. While this reduces within operator variability, it does not allow estimation of inter‑operator variability, which may be relevant for clinical shade matching procedures. Third, only one crown geometry and thickness profile were evaluated; other preparation designs or thicknesses could yield different translucency and masking behavior. Finally, the study was restricted to three A range substrate shades and did not consider more extreme discolorations (e.g. non‑vital teeth, metallic posts), which may challenge the masking capacity of highly translucent zirconias. Finally, because repeated measurements were averaged, within specimen variability could not be explored in detail.

Future studies should investigate the influence of different luting agents, ceramic thicknesses, aging procedures, and thermal cycling under more clinically relevant conditions. Studies incorporating saliva, surrounding intraoral structures, variable illumination, and clinical shade-matching procedures would further improve the translational relevance of laboratory findings.

Overall, the findings of the present study indicate that multilayer zirconia materials exhibit distinct optical characteristics and therefore cannot be considered interchangeable despite sharing the same nominal shade designation. All evaluated materials demonstrated a gradual optical transition from the cervical to the incisal region, resembling the natural tooth gradient. However, the magnitude of this gradient differed among materials, indicating that the translucency profile of multilayer zirconia systems is material dependent. In addition, the interaction between anatomical region and laboratory background conditions highlights the important influence of the underlying substrate on the final appearance of zirconia restorations, particularly in highly translucent incisal areas. From a clinical perspective, careful selection of zirconia materials together with consideration of the abutment shade and restorative design may contribute to more predictable esthetic outcomes in zirconia-based restorations.

Compared with previous studies that have predominantly evaluated optical properties or fracture behavior separately, the present study provides a comprehensive and clinically relevant analysis by simultaneously investigating material type, anatomical region, substrate shade, and laboratory background conditions. This multifactorial design allows for a more integrated understanding of the optical performance of multilayer zirconia systems and highlights the complex interactions influencing esthetic outcomes.

From a clinical perspective, material selection should be based on the desired balance between translucency and masking ability. Highly translucent materials may be preferred for achieving a natural incisal appearance when the substrate shade is favorable. In contrast, when the underlying substrate is discolored, less translucent materials or additional masking strategies should be considered. Restoration thickness and anatomical contour should also be optimized, because thinner incisal regions may enhance translucency while increasing the influence of the underlying substrate.

Given that zirconia brand, anatomical region and background shade all had statistically significant effects on ΔE₀₀ and TP₀₀ in the GLM analysis, the null hypothesis that these factors do not influence the optical properties of multilayer zirconia crowns must be rejected. Likewise, the additional null hypothesis that background shade would not affect ∆E00 within each material was rejected, as all three systems showed clinically relevant color shifts across A1, A2 and A3 substrates.

## Data Availability

The datasets generated and/or analysed during the current study are available from the corresponding author on reasonable request.
